# Elements of Sensory-Emotional Experience as an Integral Part of Forming Visual Meanings: the Role of Conceptual Abilities

**DOI:** 10.11621/pir2021.0206

**Published:** 2021-06-30

**Authors:** Marina A. Kholodnaya, Yana I. Sipovskaya

**Affiliations:** Institute of Psychology of the Russian Academy of Sciences, Moscow, Russia

**Keywords:** conceptual abilities, categorical abilities, generative abilities, visual meanings, modality, sensory-emotional experience, generative structures, embodied simulation hypothesis

## Abstract

**Background:**

This article analyzes the relationship between sensory-emotional experience in the process of semantic description of vague visual figures, and the level of conceptual (categorical and generative) abilities.

**Objective:**

The objective of our study was, first, to show the differences in the degree and features of activation of elements of sensory-emotional experience in the process of constructing the meanings of vague visual figures; and, second, to show the relationship of these differences with the level of categorical and generative abilities.

**Design:**

We studied 102 older adolescents ages 15–16 years. The research program included the following methods: 1) “Description of vague figures” (E.Yu. Artemyeva’s technique change, 1980; 1999); 2) “Generalization of three words” (Kholodnaya, 2012; Kholodnaya et al., 2019); and 3) “Conceptual synthesis” (Kholodnaya, 2012; Kholodnaya et al., 2019).

**Results:**

Our results showed that generative abilities play the leading role in determining the degree of severity and diversity of different modalities in forming visual meanings, as compared with categorical abilities. The transition simulation hypothesis explains the results. However, the embodied character of mental modeling (simulation) is not determined “bottom-up” by the individual’s bodily state or the activity of corresponding brain zones. On the contrary, conceptual (namely, generative) structures determine the form of the conceptual representations from the “top down.”

**Conclusion:**

Generative abilities represent the highest level of organization of personal conceptual experience, which acquires a multimodal quality, due to the integral nature of conceptual (generative) structures.

## Introduction

The idea of the higher verbal-logical (conceptual) forms of intellectual activity being based on sensorimotor and emotional experience has a long history. Thus, M.V. Sechenov put forward the idea that the interaction of visual (“visual crushing”) and tactile-kinesthetic (“muscle feeling”) impressions is not only the basis for the development of mental abilities in childhood, but that it also acts as a mechanism for adult conceptual thinking. “A thought constructed of symbols of any degree of generalization continues to represent a separate sensory group or sensory expression of the nervous process …” (Sechenov, 2001, p. 43). According to J. Piaget, the stage of formal thinking (“reflexive intelligence”) has its roots in a child’s sensorimotor experience, since mature intelligence has the quality of “incorporating” (integrating) all earlier forms of cognitive adaptations. That is why “… the roots of logical operations lie deeper than linguistic ties” (Piaget, 1969, p. 20).

L.M. Vekker emphasized the polymodal (intersensory) nature of thought. He noted that the manifestations of the polymodality of thinking grow to the point that they increase the degree of generalization by several levels in the process of conceptual thinking (Vekker, 1976). A series of empirical studies have supported this trend. In particular, the process of adult conceptual thinking manifests a variety of sensory-emotional impressions (visual, sound, tactile, and motor impressions with a pronounced emotional coloring), as well as varying degrees of generalization of visual images (Kholodnaya, 1974; Menshikova, 1975; Osorina, 1976).

F.E. Vasilyuk studied concepts using pictograms and concluded that “… any image, even the image associated with the abstract idea itself, is always embodied in sensitive material; it is always ‘executed’ by a whole ensemble of conscious and unconscious bodily movements and feelings” (Vasilyuk, 1993, p. 16). The reverse procedure — a verbal description of the meaning of vague visual figures — is associated with various “intermodal transitions.” Thus, “semantic-perceptual universals” have their roots in the deep structures of subjective experience (Artemieva, 1980).

There is a trend in linguistics which asserts that perceptual and sensorimotor experience is the basis for language. This view began to take shape in the early 1990s. This line of research posits a relationship between the characteristics of a person’s bodily organization and his direct interaction with his environment (*embodied cognition*, or *grounded cognition*) (Valera, Thompson, & Rosch, 1991; Barsalou, 2010; Barsalou et al., 2003; 2008; Lakoff & Johnson, 2004; Wilson & Golonka, 2013; and others). According to this approach, various aspects of sensory experience, including proprioception (*e.g*., run, lift ) and introspection (*e.g.,* hungry, happy) (Barsalou, 1999; Shallice, 1988) are the basis for high-level cognitive processes associated with the processing of verbal information. So “meaning structure” comes from “cognitive-emotional structures in a person’s mind whereby he/she makes sense of the objects and events in his/her world” (Lundh, 1995, p. 363).

This trend characterizes itself as a new and independent one (as in “the embodiment revolution began,” according to B.K. Bergen), in the absence of any references to similar, earlier psychological studies in this area.

Empirical data began to accumulate, confirming and expanding the idea that sensory-emotional experience not only plays a role in the assimilation and functioning of language, but also in the formation of the human’s conceptual sphere. In particular, cognitive linguistics has obtained empirical evidence that the concept includes sensory-perceptual, conceptual, and value elements of human experience (Maslova, 2007; Karpinets, 2004; Mahon & Caramazza, 2008; Blinnikov, 2019; and others).

The results testify to the critical role of emotional experience, even in the formation of abstract concepts. “Whereas sensorimotor information plays a central role in learning, representing, and processing concrete concepts and words, emotional information plays a central role in learning, representing, and processing abstract concepts and words” (Kousta et al., 2011). According to our research, various elements of sensory-emotional experience (in terms of 35 indicators on a modified scale of “semantic differential”) contribute to abstract concepts of a person (such as “resources” and “potential”) (Volkova & Kholodnaya, 2018). Moreover, their severity affects intellectual activity in different ways. In particular, according to the results of regression analysis, the more signs of sensory-emotional experience in the composition of a person's concepts, the lower his indicators of analytical abilities (in terms of progressive Raven matrices) and the higher the indicators non-verbal creativity as measured by Torrance's Unfinished Images tests of creative thinking.

Gradually, the idea of “multimodal” conceptual knowledge emerged, suggesting that an individual’s conceptual system participates in various modalities, such as audio, vision, touch, smell, and taste (Kibrik, 2010; Liutsko, 2013; Bruni et al., 2014; Beinborn, Botschen, & Gurevych, 2018). Experiments which visualize brain activity have reinforced this idea: the conceptual processing of information sequentially activates brain structures specific to different modalities. That is, the processes of formation and use of conceptual knowledge involve various modal systems associated with the sensory-emotional processing of information.

V. Evans formulated more subtle criteria for differences between semantic structures and conceptual structures (Evans, 2009; 2016). Conceptual structures were associated with the activation of modal systems (including sensorimotor, proprioceptive, interceptive, and affective experiences), while semantic structures functioned to offer the necessary “scaffolds” for conceptual structures.Thus, semantic structures are structures of a schematic type. By contrast, conceptual structures are structures with a wide contextual variety in the form of multimodal states.

Despite the significant differences between modern approaches to embodiment (Loginov & Spiridonov, 2017), they all focus on the idea that sensory-motor and emotional states constitute an individual’s conceptual experience “from below.”

Note that the study of elements of sensory-emotional experience as part of verbal meanings (concepts) inspired most of these studies. However, the representation of sensory-emotional experience in composing visual meanings is no less impressive, especially in the process of the semantic description of vague visual figures. Moreover, our interest was in how the degree and nature of sensory-emotional signs in the composition of visual meanings correlated with the level of formation of conceptual abilities.

In our earlier studies, the existence of different types of conceptual abilities was substantiated, including categorical and generative abilities (Kholodnaya, 2012). Categorical abilities are mental properties related to productivity of the categorization processes and to ensuring that a person will assign the corresponding object to a certain class based on transformations in the system of categorical attributes with varying degrees of generalization. Generative abilities are mental properties related to the productivity of conceptualization processes. It is an opportunity to generate some new mental constructs that are not represented in real external circumstances and are absent in the person’s acquired knowledge (*ibid*.). We set out to identify the difierent roles of categorical and conceptual abilities in enhancing sensory and emotional experience in the construction of visual meanings in this study.

Thus, the goal of this study is, first, to determine the differences in the degree and features of elements of sensory-emotional experience in the process of constructing the meaning of vague visual figures; and, second, to find the relationship of these differences with the level of formation of conceptual (categorical and generative) abilities.

### Research hypothesis

Different elements of sensory-emotional experience, activated in the process of semantic description of vague visual figures, will be differently associated with categorical and generative abilities.

## Methods

### Participants

We studied 102 older adolescents ages 15-16 years.

### Procedure

The research program included the following methods:

1) “*Description of vague visual figures,”* a modification of the E.Yu. Artemyeva method (Artemyeva, 1980; 1999).

Respondents performed two tasks when presented with vague visual figures. First, they had to answer the question: “What is it? What does this object look like?” (The respondent wrote down one or several answers). Then immediately, the respondent answered the following question: “What properties are inherent in this object, according to your impression?” (Respondents wrote down one sign or a list of signs). The following were the stimulus patterns (vague visual figures).

**Figure 1.**
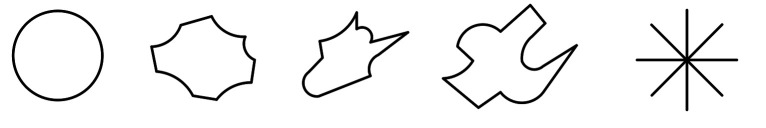
A set of vague visual figures

We identified the following indicators while assessing the respondents’ visual meanings (first task):

1. the number of meanings;
2. the number of meanings of a geometric type (*circle, ball, polygon, geometric figure, or eight triangles*);
3. the number of meanings of a subject-descriptive type (visual meaning as a direct projection of the shape of the figure: *the sun, ball, carpet, maple leaf, mask, dog’s head, cactus, hook, snowflake, or star*);
4. the number of meanings of a subject-contextual type (the meaning built on a broad, meaningful interpretation of the visual figures:*a black hole, a well, a piece of leather from a boot, a slice of cheese, a ghost, ancient weapons, a samurai, metro lines, or a schedule of functions*).

According to the instructions, the respondents, after listing the possible meanings of each figure, named a number of features by which, from their point of view, this figure can be described. The respondents named different signs that represent different modalities of experience. Based on the analysis of the protocols, we identified seven types of semantic signs which characterized different modalities of personal mental experience (second task):

- Exteroceptive modality (distant and contact), including:
  1. Visual signs (*colorful, bright, small, blue, huge, round, sparkles*, etc.);
  2. Tactile signs (*elastic, cold, soft, rough, smooth, scratchy, wet, heavy, hard*, etc.);
  3. Auditory signs (*loud, noisy, sounding, rattling*, etc.);
  4. Taste signs (*bitter, sour*, etc.) and olfactory symptoms (*pleasant smell*, etc.) (These characteristics were found in the protocols in each case in our sample. Therefore, we did not take this type of signs into account when processing our data).
- Proprioceptive modality:
  5. Proprioceptive signs based on muscle sensations during movement, *i.e.,* changes in parts of one’s body position (*running, racing, jumping, fast, tight, can explode, active, spin, fall,* etc.).
- Apperceptive modality:
  6. Apperceptive signs based on integration of sensory experience and the content of long-term semantic memory (*complex, untidy, fluid, rumpled, fragile, reliable, melts*, etc.).
- Emotional modality:
  7. Emotionally evaluative signs (*kind, ugly, cheerful, sad, affectionate, gentle, intimidating, proud*, etc.).

Indicators: 1) the total number of semantic signs mentioned in the description of the five vague visual figures, as an indicator of the activation of sensory-emotional experience;

2) the number of each of the four types of semantic signs (Exteroceptive, Proprioceptive, Apperceptive, or Emotional-evaluative) as an indicator of the severity of the different modalities; and

3) the percentage ratio of the four types of semantic signs (Exteroceptive, Proprioceptive, Apperceptive, Emotional-evaluative) on the total number of the mentioned semantic features as an indicator of the degree of severity of different ways of semantic coding.

2) “*Generalization of three words*,” a measure of categorical abilities (Kholodnaya, 2012; Kholodnaya et al., 2019).

Respondents searched for generic categories based on identifying a common essential trait between three complex concepts. We presented ten word triads, such as “*lighthouse, newspaper, bonfire*;” “*icon, map, decoration;*” “*gamma, beads, stairs*,” etc.

Indicator: the sum of the scores for 10 triads of words as an indicator of categorical abilities. Evaluation criteria for each answer: 0 points - thematic generalization (theater, tourists, childhood, etc.); 1 point - analytical generalization (*built by a man, many details, can give light, long, etc*.) or formal generalizations without highlighting an essential feature (*labor, nature, decoration, artificial object, etc*.); 2 points - categorical generalization (*sources of information, structures, images, sequence, etc*.).

3) “*The conceptual synthesis*,” a measure of generative abilities (Kholodnaya, 2012; Kholodnaya et al., 2019).

Respondents composed sentences combining three unrelated words based on generating their own context. We presented three word triads, such as “*shell, paper clip*, *thermometer*;” “*computer, tornado, pin*;” “*planet, hourglass, electrical outlet*.”

Indicator: the sum of the scores for all completed sentences for the three triads of words as an indicator of conceptual ability. Evaluation criteria for each answer: 0 points — only two words out of three are connected, or a meaningless combination of words is formed; 1 point — the link is established on the basis of a simple listing of three words without specifying the links between them; 2 points — all three words are included in the description of a specific situation; 3 points — all three words are connected on the basis of cause-and-effect relationships, generalizing categories, metaphors, combining different contexts.

We use a standardized IBM SPSS software package (version 26) for data processing. Previously, we had normalized all indicators.

## Results

### Correlation Analysis

First of all, we analyzed the correlations (according to Spearman) between the types of visual meanings and the types of semantic signs, since the kind of visual meanings the respondents created during the first task determined their selection of semantic attributes: respectively, sensory and emotional experience activation. The results were as follows.

The total number of named visual meanings was associated with the total number of selected semantic signs (r = 0.315, p = 0.001). In turn, there were no associations between any type of semanticsigns and the number of geometric meanings. The number of subject-descriptive meanings was definitely associated with the number of Exteroceptive (r = 0.265, p = 0.007) and Proprioceptive (r = 0.219, p = 0.03) signs, while the number of subject-contextual meanings was associated with the number of Proprioceptive (r = 0.230, p = 0.02) and Emotional-evaluative (r = 0.348, p = 0.000) signs. These relationships relate to the core subject of our research, the process of constructing visual meanings (both their actualization and the allocation of their individual semantic features).

Next, we analyzed the relationship between indicators of the severity of different types of semantic signs and indicators of the formation of categorical and generative abilities.

According to the correlation analysis (according to Spearman), only indicators of generative abilities are definitively associated with all indicators of the severity of semantic signs without exception: the total number of signs (r = 0.461, p = 0.000); the number of Extraceptive signs (r = 0.300, p = 0.002), including the number of visual (r = 0.249, p = 0.012), tactile (r = 0.250, p = 0.011), or auditory (r = 0.271, p = 0.006) signs; the number of Proprioceptive signs (r = 0.387, p = 0.000); the number of Apperceptive signs (r = 0.241, p = 0.015); and the number of Emotional-evaluative signs (r = 0.392, p = 0.000). Categorical abilities have one weak connection with the number of Emotional-evaluative signs (r = 0.215, p = 0.030).

### Factor Analysis (Principal Component Method; Varimax Rotation Method with Kaiser Normalization).

*Table 1* shows a factor matrix. It includes indicators of categorical and generative abilities, as well as measures of the severity of Extraceptive, Proprioceptive, Apperceptive, and Emotional-evaluative signs. The KMO value is 0.679 and the significance level of the Bartlett sphericity criterion for both samples is p = 0.000. Eigenvalues of the factors are more than one; total dispersion is 57.0%.

**Table 1:** Factorization results (after rotation) of conceptual abilities and the main types of semantic signs

Components	1 factor (39.5%)	2 factor (17.5%)
Categorical abilities	–.025	.929
Generative abilities	.531	.551
Extraceptive signs	.560	.108
Proprioceptive signs	.782	–.130
Apperceptive signs	.690	.134
Emotional-evaluative signs	.632	.354

According to *Table 1*, although categorical and generative abilities interconnect (2 factors), nevertheless, all four types of semantic signs were associated with only one type of conceptual ability, namely the indicator of generative abilities (1 factor). Therefore, we can say that sensory-emotional, experience is activated in the process of semantic description of vague visual figures, and is associated primarily with conceptual generative abilities.

### Cluster Analysis

The first variant of cluster analysis was associated with the identification of subgroups of respondents simultaneously according to two criteria: the level of formation and categorical and conceptual abilities (all indicators have a normal statistical distribution). *Table 2* presents the three clusters that stood out, and their characteristics.

**Table 2:** Descriptive characteristics of clusters identified by the criteria for categorical and generative abilities formation

Clusters	The number of subjects	Categorical abilities	Generative abilities
1	20	–0.52 ± 0.59	1.00 ± 0.57
2	58	–0.38 ± 0.72	–0.65 ± 0.50
3	24	1.34 ± 0.57	0.73 ± 1.00

*Table 2* shows that cluster 2 is a subgroup of respondents with a low level of conceptual abilities (both categorical and generative). Cluster 3 is a subgroup of respondents with high-level conceptual abilities (both categorical and generative). Cluster 1 is a subgroup with an imbalance of conceptual abilities in the form of a pronounced predominance of generative abilities over categorical abilities. We compare these subgroups with each other in terms of measures of the severity of the main types of semantic attributes: Exteroceptive, Proprioceptive, Apperceptive, and Emotional-evaluative.

Respondents with a high level of conceptual abilities (cluster 3) identified more Apperceptive (p = 0.05) and Emotional-evaluative (p = 0.007) signs than respondents with low-level conceptual abilities (cluster 2). In other words, a higher level of conceptual abilities leads to a more pronounced activation of Apperceptive and Emotional-evaluative signs.

On the other hand, respondents with an imbalance of conceptual abilities (cluster 1), as compared with respondents with a low level of conceptual abilities (cluster 2), chose more Extraceptive (p = 0.05) and Emotional-evaluative (p = 0.05) signs. Consequently, activation of Extraceptive and Emotional-evaluative signs appears in the case of the predominance of generative abilities, combined with low-level categorical abilities.

The second version of the cluster analysis involved classifying the sample according to the severity criteria of all four main types of semantic signs: the percentages of Extraceptive, Proprioceptive, Apperceptive, and Emotional-evaluative (all indicators have normal distribution). The analysis allowed us to name three clusters; *Table 3* presents their characteristics.

**Table 3:** Descriptive characteristics of clusters, identified by the criteria for the percentage of four types of semantic signs

Clusters	% Extraceptive signs	% Proprioceptive signs	% Apperceptive signs	% Emotional-evaluative signs
1 (n=67)	0.55	–0.04	–0.12	0.34
2 (n=22)	–1.27	–0.51	–0.73	–0.79
3 (n=13)	–0.70	1.06	1.84	–0.45

According to *Table 3*, cluster 2 is a subgroup of respondents with low indicators of the severity of all types of semantic attributes. Respondents with the predominance of Exteroceptive and Emotional-evaluative signs belong to cluster 1. Cluster 3 consists of respondents with a predominance of Proprioceptive and Apperceptive signs. Then we compare subgroups among themselves in terms of their categorical and generative abilities.

We found significant differences in the indicator “level of formation of generative abilities” (p = 0.000) when comparing cluster 2 and cluster 1 (indicators of generative abilities are higher among respondents of cluster 1). At the same time, there were no differences in the indicator “level of formation of categorical abilities” when comparing cluster 2 and cluster 1.

Similarly, we noted significant differences in the indicator “level of formation of generative abilities” (p = 0.003), when comparing cluster 2 and cluster 3 (indicators of generative abilities are higher among respondents of cluster 3). However there were no differences in the indicator “level of formation of categorical abilities” when comparing cluster 2 and cluster 3.

Thus, according to the results of cluster analysis, we can draw the following conclusion: A higher level of conceptual abilities (both categorical and generative) is associated with a more pronounced activation of Apperceptive and Emotional-evaluative signs (the first version of cluster analysis). Nevertheless, generative abilities play a leading role in activating sensory and emotional experience in the process of semantic description of vague visual figures. A high level of generative abilities implies a higher severity of both Exteroceptive and Emotional-evaluative, as well as Proprioceptive and Apperceptive modalities in the composition of visual meanings (the second variant of cluster analysis). Judging by our data, categorical abilities are not a determining factor in activating an individual’s sensory-emotional experience.

### Network Analysis

We carried out a network analysis to confirm that the elements of sensory-emotional experience are associated with the level of formation of generative abilities. We used analysis of a weighted network of correlations (a weighted network of gene co-expression, WGCNA or network analysis) to cut the number of variables without losing the significant relationships evident in in-depth data analysis. The method allowed us to define modules (clusters), intermodal hubs, and network nodes on module membership, and find relationships between modules and compare topologies of different networks.

The network itself has the form of a graph composed of nodes connected by edges (connections between nodes) and indicating the “weights” of the edges of the network. The sign of the edge weight (positive or negative) indicates the type of interaction, and the absolute value of the edge weight indicates the strength of the connection between the nodes (Fruchterman et al., 1991; Newman, 2010; Opsahl, Agneessens & Skvoretz, 2010). The use of network analysis has shown its worth in the analysis of indicators of the productivity of intellectual activity (Sipovskaya, 2019; Sipovskaya, 2020).

**Figure 2.**
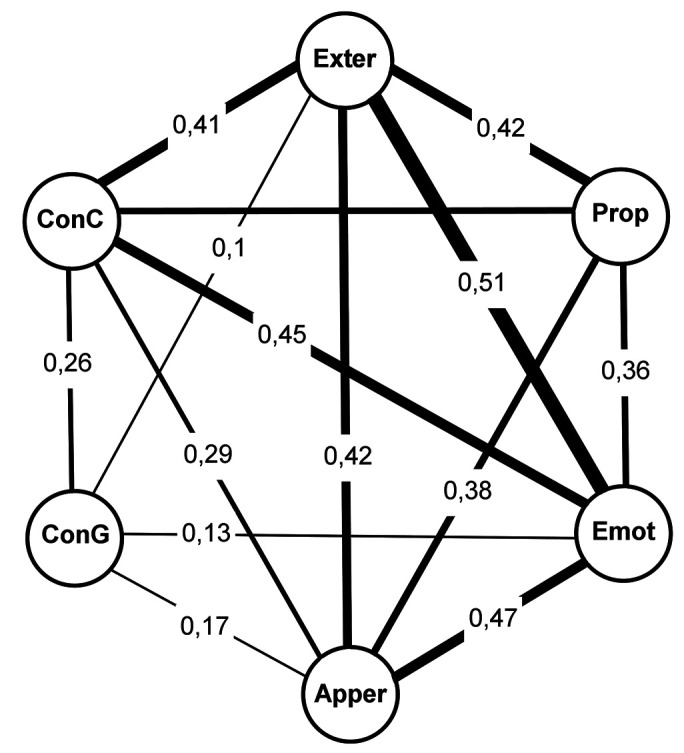
Modeling the structure of conceptual experience (elements of sensory-emotional experience in a system of relations with categorical and generative abilities)

Note. ConC = categorical abilities; ConG = generative abilities; Exter = Exteroceptive signs; Apper ± Apperceptive signs; Emot = Emotional-evaluative signs; Prop = Proprioceptive signs.

*Figure 2* presents the linkage of elements of sensory-emotional experience with the level of formation of categorical and generative abilities through network modeling.

*Figure 2* shows us that generative abilities are associated with all four types of semantic attributes. It is noteworthy that Extraceptive (0.41) and Emotional-evaluative (0.45) signs have the most robust connections with conceptual generative abilities. In turn, there are three types of semantic signs associated with categorical conceptual abilities. However, all three connections are weak, not reaching an acceptable level of significance (0.10; 0.13; 0.17).

Thus, the results of network analysis confirm that, on the one hand, the level of formation of conceptual abilities (both categorical and generative) is associated with their measure of participation in the process of semantic description of vague visual figures, and many elements of sensory and emotional experience. On the other hand, we may consider only conceptual generative abilities as the leading factor in the activation of sensory-emotional experience in this type of intellectual activity.

## Discussion

According to different types of data processing — correlation, factorial, cluster, and network analysis — conceptual generative abilities, unlike categorical conceptual abilities, involve elements of sensory-emotional experience in the process of semantic description of vague visual figures.

The question arises: Why were only generative abilities associated with the activation of sensory-emotional experience? We emphasize that it is important not to confuse generative (conceptual) abilities with general abilities, which are traditionally described in terms of IQ indicators.

We define generative abilities operationally as the ability to create new mental contexts when constructing “impossible connections” between three concepts that differ in meaning. Accordingly, we can turn to the embodied simulation hypothesis to explain the unique role of generative abilities in the activation of sensory-emotional experience. Simulation is the creation of mental products (in the form of personal constructs, representations of certain situations, mental actions, etc.) in the absence of external stimulation. In other words, the embodied simulation hypothesis states that meaning is what a person creates in their mind based on their own mental experience, including the activation of its sensory and emotional components. “Meaning, according to the accomplished simulation hypothesis, isn’t just abstract mental symbols; it’s a creative process, where people construct virtual experiences — implemented simulations — in their mind’s eye” (Bergen, 2012, p. 22). It is not surprising that generative abilities, according to various forms of our data analysis, are directly related to the activation of sensory and emotional experience.

In other words, we are talking about the specific quality of intelligence called mental modeling (simulation) and associated with the level of formation of conceptual (primarily generative) abilities, *i.e*., the creation — in the absence of an external stimulus — of mental constructions (“mental images” and “mental actions”), the mental “material” of which is sensory and emotional impressions. No wonder that it is generative abilities that are associated with the intensity and variety of elements of sensory and emotional experience - a kind of construct that can create the meanings of vague visual images.

Finally, the main question: What is the source of these sensory-emotional impressions, and what mental mechanism is responsible for their activation?

In our opinion, the source that generates and regulates the influx of sensory-emotional impressions into intellectual activity (in our case, into the process of construction of visual meanings) is generative structures as mental units of personal conceptual experience. Generative structures are integral cognitive formations that contain a system of multilevel information-processing mechanisms. Generative structures are integral cognitive formations that contain a system of multilevel information-processing mechanisms. They include effective means of sensory, motor, and emotional coding, visualization, placement in semantic networks, categorization, generation of new mental contents, etc. (Kholodnaya, 2012). A higher degree of formation of generative structures leads to a higher level of conceptual abilities and, so, there will be more elements of sensory and emotional experience in conceptual representations.

Thus, mental modeling (simulation) has an embodied character not in the sense that it is determined “bottom-up” by bodily states or the corresponding activity of certain brain zones. On the contrary, the functioning of generative structures determines how conceptual representations are embodied “from the top.” Since in conceptual structures, as they are formed, the experience of the interaction of the body (including the brain) with its environment is accumulated, generalized and integrated, which later appears in various effects of embodied cognition.

## Conclusion

An analysis of how activation of elements of sensory-emotional experience in the semantic description of vague visual figures relates to the level of formation of categorical and generative abilities, allowed us to draw the following conclusions: 1) Generative abilities play the leading role in the degree of severity and degree of diversity of different modalities in visual meanings composition; and 2) Generative abilities characterize the highest level of organization of personal conceptual experience, which, due to the integral nature of conceptual (generative) structures, takes on a multimodal quality.

## Limitations

Two factors may limit the generalizability of our results. First, the specifics of the sample:Older adolescents are experiencing the peak of development of their conceptual abilities at this age. Accordingly, it is necessary to check the severity of this effect — the multi-directional role of categorical and generative abilities — in an adult sample. Second, the specifics of intellectual activity in the form of formulating visual meanings. The question arises as to whether the same effect would be evident if the task involved verbal meanings.
